# Toxicology

**Published:** 1836-07

**Authors:** 


### TOXICOLOGY.

### *Experiments on the Action of Oxalic Acid.* By J. W. Arnold.

These experiments were performed on dogs, cats, birds, and (chiefly) on rabbits: in the latter, the acid does not produce vomiting, and consequently it is unnecessary to tie the œsophagus. The poison was introduced into the stomach by pouring it slowly into a horn funnel attached to the outer end of an elastic tube passed down the gullet,—a mode of proceeding which makes it unnecessary to confine the animal.

The following are the general conclusions drawn from the experiments made with acid prepared and administered under various circumstances.

1. Oxalic acid is an active poison, and when given in large doses usually proves quickly fatal.

2. Its poisonous effects are uniform, and independent of extraneous admixtures, the mode of preparation, &c.

3. Its immediate effect on the nervous system is stimulant. This primary excitement is more or less rapidly succeeded by diminished vigour of the nervous functions.

4. The effect upon the heart is of the same kind, and dependent upon that produced on the nervous system; it occurs very speedily,—by a very transient state of extraordinary excitement being succeeded by diminution and ultimately by cessation of the heart's action. Similar observations are applicable to the respiratory organs.

5. The blood is peculiarly affected by oxalic acid. After large and speedily fatal doses it is fluid, uncoagulable; which is not the case after smaller and less rapidly destructive doses. This action cannot be ascribed to a chemical solution, as is sufficiently proved by its succeeding only to a quick, almost sudden death from the poison. Further, the coagulum of blood macerated at an elevated temperature, for a month together, in a solution of the acid, is not affected in this manner. It may consequently be assumed that the blood is thus affected by the acid as a result of the action of the latter on the nervous system; a supposition that will not be rejected by those physiologists, at least, who consider the blood as capable of being immediately influenced by the nervous system. The great accumulation of the blood in the venous portions of the heart and vascular system is to be explained by the impaired state of respiration and circulation. Like other acids, as Stevens and Hartwig have shewn, the oxalic renders the colour of the blood dark.

6. As regards the action on the alimentary canal, it operates as a local irritant, causing inflammation and extravasation of blood,—effects which are less obvious in proportion to the rapidity with which death ensues. Black points and patches occur on the mucous surface in consequence of the chemical action on the effused blood. The solution of the mucous membrane appears also to depend on chemical causes, inasmuch as it is always more decided in proportion to the interval which elapses between death and the time of examination.

7. The best means of counteracting the operation of the poison are those which tend to its evacuation; among which, emetics, however, are the less to be recommended, as the vomiting produced by the poison itself has little efficacy in preventing its injurious consequences. The stomach-pump is the most certain, if it can be used early enough. The employment of such bases as unite to form soluble salts with the acid, is of no service, as such salts are themselves poisonous. Magnesia and lime, on the contrary, deserve attention, as their combinations with the acid are insoluble. Stimulants, such as alcohol and camphor, have been proposed to counteract the depressing effects, particularly on the nervous system: but the rapidity of a fatal termination from large doses of the poison usually renders all aid useless.

8. Lastly, these experiments prove, what had been already rendered probable by observations on the human subject in health and disease, that neither the dilatation nor contraction of the pupil is to be considered as a simply passive condition.

*Tiedemann's Zeitschrift für Physiologie.* Vol. V. 1835.

